# Labour force participation and the cost of lost productivity due to cancer in Australia

**DOI:** 10.1186/s12889-018-5297-9

**Published:** 2018-04-06

**Authors:** Nicole Bates, Emily Callander, Daniel Lindsay, Kerrianne Watt

**Affiliations:** 1 College of Public Health, Medical and Veterinary Sciences, James Cook University, Building 48, Douglas Campus, Townsville City, Queensland 4811 Australia; 2 Australian Institute of Tropical Health and Medicine (AITHM), James Cook University, Townsville, Queensland 4811 Australia

**Keywords:** Cancer, Oncology, Costs, Health economics, Productivity

## Abstract

**Background:**

In Australia, 40% of people diagnosed with cancer will be of working age (25–64 years). A cancer diagnosis may lead to temporary or permanent changes in a person’s labour force participation, which has an economic impact on both the individual and the economy. However, little is known about this economic impact of cancer due to lost productivity in Australia. This paper aims to determine the labour force participation characteristics of people with cancer, to estimate the indirect cost due to lost productivity, and to identify any inequality in the distribution of labour force absence in Australia.

**Methods:**

This study used national cross-sectional data from the 2015 Survey of Disability, Ageing and Carers, conducted by the Australian Bureau of Statistics (ABS). The ABS weighted each component of the survey to ensure the sample represented the population distribution of Australia. The analysis was limited to people aged 25–64 years. Participants were assigned to one of three health condition groups: ‘no health condition’, ‘cancer’, and ‘any other long-term health condition’. A series of logistic regression models were constructed to determine the association between health condition and labour force participation.

**Results:**

A total of 34,393 participants surveyed were aged 25–64 years, representing approximately 12,387,800 Australians. Almost half (46%) of people with cancer were not in the labour force, resulting in a reduction of $1.7 billion to the Australian gross domestic product (GDP). Amongst those in the labour force, people with no health condition were 3.00 times more likely to be employed full-time compared to people with cancer (95% CI 1.96–4.57), after adjusting for age, sex, educational attainment and rurality. Amongst those with cancer, people without a tertiary qualification were 3.73 times more likely to be out of the labour force (95% CI 1.97–7.07).

**Conclusions:**

This paper is the first in Australia to estimate the national labour force participation rates of people with cancer. People with cancer were less likely to be in the labour force, resulting in a reduction in Australia’s GDP. Cancer survivors, especially those without a tertiary qualification may benefit from support to return to work after a diagnosis.

## Background

Cancer is the leading burden of disease in Australia [1] and internationally [2]. In 2017, an estimated 134,174 Australians will be diagnosed with cancer, of which 40% will be of working age (25–64 years) [3]. Using the 2003 Survey of Disability, Ageing and Carers, Schofield et al. [4] found that half (49%) of older Australians (aged 45–64 years) with cancer were not in the labour force. In a more recent Australian study of 255 cancer patients, 67% reported changes to their employment [5]. A systematic literature review of employment and work-related issues in cancer survivors included 64 international studies and found that over a six-year period after a cancer diagnosis, 26–53% of cancer survivors were out of work (lost their job or quit) [6]. Similarly, a recent meta-analysis of international studies found that compared to healthy control participants, cancer survivors were 1.4 times more likely to be unemployed [7]. These studies focus on the individual perspective, and highlight the important financial distress faced by individual cancer patients and their families due to being out of the labour force [5, 8–12]. However, to date there has been minimal focus on the societal perspective in Australia, and the consideration of the aggregate costs of these changes in workforce participation; nor has there been consideration of the distribution of the costs of labour force absence, looking at the population groups most likely to be affected. Such information could be more useful to policy makers, concerned with maximising welfare to the whole of society.

Health economic evaluations routinely evaluate the direct cost of illness; however, the indirect cost of lost productivity due to morbidity and premature mortality may exceed the direct costs of cancer [13–17]. In Europe, the estimated cost of lost productivity from cancer in 2009 was 42%, a further 18% was attributed to the cost of informal care, and only 41% of costs were attributed to direct health care [18]. The National Institute of Health estimated that in 2010, the cost of lost productivity in the United States accounted for 61% of the total cost of cancer, compared to 39% for the direct costs [15]. In Korea, the estimated morbidity and premature mortality costs accounted for 55% of the total cost of cancer, compared to 28% for medical care in 2009 [17]. In New South Wales, Australia in 2005, lost productivity accounted for 54% of the total lifetime cost of cancer, compared to the direct costs which accounted for 29% of the total lifetime costs [13]. However, this report was from a single state in Australia. Carter, Schofield and Shrestha [19] recently estimated that approximately 88,000 working years were lost, due to premature deaths from cancer in 2003 in Australia, which cost $4.2 billion in present value of lifetime income. Lung and colorectal cancers accounted for 30% of the total loss of income. This study provides a national perspective of the cost of cancer due to premature mortality; however, there is currently limited work on the productivity cost for people with cancer who are out of the labour force.

This paper will contribute to the growing body of research on the indirect costs of cancer. The aims of this research conducted within an Australian context are to: 1) determine whether people with cancer have different labour force participation characteristics to people with no health conditions, or any other long term health conditions, 2) estimate the cost of cancer due to lost productivity (limited to the context of paid work) and 3) identify any inequality in the distribution of labour force absence amongst those with cancer.

## Methods

### Data

The primary data source accessed for this data was the 2015 Survey of Disability, Ageing and Carers (SDAC). This is a national survey conducted every 3 years by the Australian Bureau of Statistics (ABS). The SDAC sample was a randomly selected sample of the Australian population, regardless of health condition or disability status and included participants from both household and cared-accommodation, but excluded those residing in prisons/correctional institutes, religious and/or educational institutes; very remote areas, and discrete Indigenous communities. The sample frame comprised approximately 25,500 private dwellings, 250 self-care retirement, and a further 1000 cared-accommodation facilities. To adjust for any potential bias in survey participants, the ABS weight the survey data against known population benchmarks. Briefly, the weight value indicates how many of the population unit each sample unit represents. The two components were weighted separately, the household component was benchmarked to the estimated resident population in each jurisdiction, and the cared accommodation component was benchmarked to the census population counts of this component as described in detail by the ABS [20]. Weighting allows an inference of results to the general Australian population.

The survey included questions on demographics (such as age, sex, highest level of education achievement, and geographical remoteness), labour force participation, and long-term health conditions (LTHC). LTHC’s were defined by the SDAC as a condition lasting, or was likely to last 6 months or more, or symptoms in the previous 12 months for an episodic condition (ie asthma or epilepsy) and coded by the ABS based upon the ICD-10 [20]. Although the original survey included answers for specific medical conditions, the ABS regrouped some conditions for data release. For example, the types of cancer were grouped by the ABS: skin cancer (ICD C43–44), breast cancer (ICD C50), prostate cancer (C61), bowel/colorectal cancers (C18–21) and any other neoplasm (including benign tumours).

### Statistical analysis

Analyses were limited to people aged 25–64 years. Excluding survey respondents under the age of 25 allowed those most likely to be participating in higher education to be excluded; and those over the age of 65 were excluded, as the traditional retirement in Australia in 2015 was 65 years. Participants were categorised into one of three health condition groups: ‘no LTHC’ included those who did not report any LTHC, ‘cancer’ included those who reported having cancer (skin, breast, prostate, bowel/colorectal or any other neoplasm) as a LTHC, and ‘any other LTHC’ included those who reported any other LTHC. Labour force participation (LFP) was recorded as employed full-time (FT), employed part-time (PT), unemployed but looking for FT and/or PT work, and not in the labour force (NILF).^1^ Educational attainment was recoded into a dichotomous variable (tertiary vs non-tertiary education); as was rurality (major cities and other), and age (25–44 years and 45–64 years).

### Aim 1: Labour force participation of people with cancer

A series of logistic regression models were constructed to determine the association between cancer and LFP. Initially, the analysis was limited to include those in the labour force only. A logistic regression model was constructed to estimate the odds of being employed full-time for people with cancer, LTHC, and no health condition (respectively), after adjusting for age, sex, educational attainment and rurality. A second logistic regression model was constructed to estimate the odds of being out of the labour force for people with cancer, LTHC, and no health condition (respectively), after adjusting for age, sex, educational attainment and rurality.

### Aim 2: Cost of labour force absence associated with cancer

Using the same approach as other Australian studies [21–25], the financial impact to Australia’s gross domestic product (GDP) due to people with cancer being out of the labour force was estimated using the Australian Treasury’s formula [26]:

$$ \mathsf{GDP}=\left(\mathsf{GDP}/\mathsf{H}\right)\ \mathsf{x}\ \left(\mathsf{H}/\mathsf{EMP}\right)\ \mathsf{x}\ \left(\mathsf{EMP}/\mathsf{LF}\right)\ \mathsf{x}\ \left(\mathsf{LF}/\mathsf{Pop}\mathsf{15}+\right)\ \mathsf{x}\ \mathsf{Pop}\mathsf{15}+ $$

where GDP = gross domestic product; H = total hours worked; EMP = total number of persons employed; LF = total labour force; and Pop15+ = population aged 15 years and over [26].

This method has been previously used in other studies [21–25], and differs from the friction cost method, which argues that people who leave the labour force due will be replaced by other workers (including those who were previously unemployed) thus limiting the cost of workers leaving the labour force [27]. Australia has a very low unemployment rate (6.3% in July 2015) [28] and significant labour shortages in some industries [29], furthermore, the Australian Treasury’s aim to make Australia’s financial position more sustainable by promoting productivity, population growth and labour force participation [30], recognising the signficiant cost labour force exit has on the Australian economy.

### Aim 3: Inequality in the distribution of labour force absence amongst those with cancer

A concentration index was initially used to determine whether there was any inequality in the distribution of labour force absence amongst people with cancer. The concentration index is normally used as a measure of health inequality, and assesses the distribution of health outcomes across socioeconomic groups in a population. The concentration index reflects the cumulative proportion of health held by the cumulative proportion of the population, ranked by a measure of socioeconomic status. The measure of socioeconomic status used was the highest level of education attainment achieved, as reported in the survey (7 ordinal categories: Year 8 or below, Year 10, Year11/12, Certificate, Diploma, Bachelor, Post-graduate). The concentration index ranges from − 1 to 1, with a value of 0 denoting perfect equality in the distribution of labour force absence, a negative value denoting a distribution skewed towards people of lower socioeconomic status, and a positive value denoting a distribution skewed towards people of higher socioeconomic status. The concentration index (CI), and it’s associated 95% confidence intervals, were computed as follows:

$$ 2{\sigma}_R^2\left(\frac{y_i}{\mu}\right)=\alpha +\beta {R}_i+{\varepsilon}_i $$

Where ${\sigma}_R^2$ is the variance of *R*_*i*_ (the individual’s rank), *y*_*i*_ is the labour force status of each individual (*i* = 1, 2, 3….N), *α* is the intercept, *ε*_*i*_ is the error terms, and *β* is the CI ^1^. Finally, among only people with cancer, a multivariate logistic regression model was constructed to estimate the odds of being not in the labour force, after accounting for age, sex, educational attainment, and rurality.

All analyses were undertaken using SAS V9.4 (SAS Institute Inc., Cary, NC, USA). Weighted estimates are presented, unless stated otherwise. GDP figures are presented in 2015 Australian dollars.

## Results

Within the 2015 SDAC, a total of 34,393 participants were of working age (25–64 years), which represented approximately 12,387,800 people when weighted. Of the participants in this age group, there were 7,287,100 with no health conditions, 108,900 people with a type of cancer, and 4,991,800 with some other LTHC. Table 1 shows the demographic characteristics for each of the health condition categories (no LTHC, cancer, and any other LTHC).

**Table 1:** SDAC sample demographic characteristics of Australian adults of working age, 25–64 years (using weighted totals, rounded to the nearest 100)

	Long-term health condition		
	No LTHC Total (%)	Cancer Total (%)	Any other LTHC Total (%)
Total			
Survey Participants	18,990	355	15,048
Weighted Population estimate	7,287,100	108,900	4,991,800
Sex (weighted)			
Male	3,641,800 50%	46,600 43%	2,420,400 48%
Female	3,645,300 50%	62,300 57%	2,571,500 52%
Age (weighted)			
25–44 years	4,615,600 63%	18,500 17%	1,960,200 39%
45–64 years	2,671,500 37%	90,400 83%	3,031,700 61%
Educational attainment (weighted)			
Non-tertiary	4,429,000 62%	75,500 71%	3,526,200 73%
Tertiary	2,702,000 38%	30,100 29%	1,336,700 27%
Rurality (weighted)			
Major cities	5,610,800 77%	74,500 68%	3,425,500 69%
Other areas	1,676,200 23%	34,400 32%	1,566,300 31%

*LTHC* long-term health condition

### Aim 1: Labour force participation of people with cancer

Table 2 shows the employment status for people with no health condition, people with cancer, and people with any other LTHC. Almost half (46%) of people with cancer were not in the labour force, compared to approximately a quarter (27%) of people with any other LTHC, and only 12% of people with no health condition.

**Table 2:** Labour force participation of participants (weighted)^a^

Health condition	Employed FT Number (%)	Employed PT Number (%)	NILF Number (%)	Total weighted population estimate
No LTHC	4,585,900 62.9%	1,589,700 21.8%	887,100 12.2%	7,287,000
Cancer	27,300 25.3%	30,400 28.1%	50,100 46.4%	108,100
Any other LTHC	2,328,700 46.8%	1,104,900 22.2%	1,344,100 27.0%	4,975,900

*LTHC* long-term health condition, *FT* full-time, *PT* part-time, *NILF* not in the labour force, have not looked for work in the last 4 weeks, and do not intend to work or look for work in the future

^a^The number and percentage of people who were ‘unemployed’ were not presented due to low unemployment rate in Australia, and hence the low sample number of unemployed people

Firstly, the analyses were limited to people who were in the labour force. Of those with cancer who were employed, 47% were employed full time, compared with 68% of those with any other LTHC, and 74% of those with no health condition. Amongst those in the labour force, after adjusting for age, sex, educational attainment and rurality, those with no health condition had 3.00 times the odds of being employed full-time than people with cancer (95% CI 1.96–4.57; *p* <  0.0001). Similarly, those with any other LTHC had 2.15 times the odds of being employed full-time (95% CI 1.41–3.28; *p* = 0.0004) than those with cancer.

Secondly, the odds of being out of the labour force were calculated. Table 3 shows that after adjusting for age, sex, educational attainment and rurality, those with no health condition, and those with any other long-term health condition had lower odds of being out of the labour force compared to adults with cancer.

**Table 3:** Logistic regression model of being not in the labour force

*Parameter*	*Estimate*	*Standard Error*	*P-Value*
Intercept	−2.49	0.16	< 0.001
Male	0.97	0.35	< 0.001
Aged 25–44	0.35	0.03	< 0.001
Tertiary education attainment	0.70	0.04	< 0.001
Lives in major city	0.04	0.04	0.2255
No LTHC	−1.68	0.14	< 0.001
Any other LTHC	−0.81	0.14	< 0.001
Odds of being out of the labour force			
	Odds Ratio^a^	95% CI	*P*-value
Cancer	Reference		
No LTHC	0.19	0.14–0.25	< 0.001
Any other LTHC	0.45	0.34–0.58	< 0.001

*LTHC* long-term health condition

^a^adjusted OR = adjusted for age, sex, educational attainment, and rurality

### Aim 2: Cost of labour force absence associated with cancer

An estimated 50,100 Australian adults of working age (25–64 years) with cancer were not in the labour force in 2015, thereby reducing Australia’s GDP by approximately $1.7 billion (Table 4).

**Table 4:** The proportion of people out of the labour force, and the financial impact on Australia’s GDP amongst people with cancer (weighted)

	Estimated number NILF	Total people with cancer	% NILF/total people with cancer	Difference in GDP AU$ million
Total				
	50,100	108,900	46%	$1738 million
Sex				
Male	19900^a^	46,600	43%	$690 million
Female	30300^a^	62,300	49%	$1051 million
Age				
25–44 years	6900	18,500	37%	$239 million
45–64 years	43,200	90,400	48%	$1499 million
Educational attainment				
Non-tertiary	42,100	75,500	56%	$1460 million
Tertiary	7500	30,100	25%	$260 million
Rurality				
Major cities	32,100	74,500	43%	$1114 million
Other areas	18,000	34,400	52%	$624 million

^a^rounded up

### Aim 3: Inequality in the distribution of labour force absence amongst those with cancer

The concentration index showing level of inequality in the distribution of labour force absence amongst people with cancer was calculated to be − 0.20 (95% CI -0.26 to − 0.13), which indicates that having cancer and being not in the labour force is unequally skewed towards those with a low educational attainment.

Amongst people with cancer, those without a tertiary qualification were 3.73 times more likely to be out of the labour force (95% CI 1.97–7.07; *p* <  0.0001), than people with tertiary education. Sex, age, and rurality were not associated with being out of the labour force (*p* = 0.0818, *p* = 0.3723, *p* = 0.1869 respectively).

## Discussion

The results of this paper have shown that in 2015, almost half (46%) of adults of working age (25 to 64 years) with cancer were not in the labour force. Of those in the labour force, adults with no health conditions had 3 times the odds of being employed full-time than adults with cancer. Other studies have supported our findings that following a cancer diagnosis, many patients report a temporary or permanent change to their labour force participation, including a reduction in work hours and stopping work [5, 6, 8, 9, 11, 31–34].

Amongst people with cancer, a greater proportion of older people (45–64 years) were not in the labour force, however, this appears to be explained by other demographic factors. Amongst people with cancer, those without a tertiary qualification had nearly four times the odds of being out of the labour force; age, sex, and rurality were not associated with a greater risk. Higher education attainment has been identified as a positive factor in returning to work [6, 35], which also supports our findings that labour force absence amongst those with cancer is unequally skewed towards those with lower levels of education attainment. Other factors which may affect returning to work include jobs that require manual labour, and those with less flexible working arrangements [6, 8, 10, 32, 33]. The type of cancer and treatment factors may also negatively impact returning to work [6, 7, 10, 35]. For example, Clarke et al. [35] found that colorectal and lung cancer survivors had greater difficulties performing daily activities, and colorectal, lung, and bladder cancer survivors were more likely to have functional limitations, which may impact work ability.

This paper looked at distribution of people being out of the labour force for any reason, with some people potentially being out of the labour force directly as a result of cancer, while others may have chosen early retirement [11] due to a reassessment of their priorities following a health shock. In Australia, employees (excluding casual employees) are entitled to paid sick leave (10 days each year for full-time employees), and unpaid leave for up to 3 months [36]. While the Australian retirement age in 2015 was 65 years, at which time individuals may be eligible to access a government ‘age pension’, individuals may have access to their superannuation from age 55. However, previous work has found that despite these sources of financial support, early retirement due to LTHC has a significant negative impact on an individual’s income and wealth [22, 25]. As such cancer survivors may benefit from additional support by the Government, employers and medical professionals to facilitate returning to work if they choose to. Previous studies have shown that many cancer survivors will return to work after treatment, from 40% at six-months to 89% at 24-months following a cancer diagnosis [6].

A key strength of this paper is that it used the 2015 SDAC, which is a national survey weighted to the Australian population. Participants who identified as having cancer as a long-term health condition represented 0.9% of the weighted population. It was estimated that around 1.5% of the Australian population had cancer in 2011–12 [37]. However, we limited our analyses to people of working age only (25–64 years), which excluded people over 75 years, who had the highest rate of developing cancers (11.1% for men and 4.4% for women) [37]. Therefore, this study was representative of the Australian population of working age.

However, the use of the SDAC also has several limitations. This survey did not include information about the time since diagnosis, which may be a factor in labour force participation [8, 9, 12]. Secondly, although the main types of cancer in Australia (skin, breast, prostate, and colorectal cancer) are identified in the SDAC, the number of participants in each type was small, and therefore, disaggregated analysis by type of cancer was not possible. Finally, it is possible that some types of cancers, which may have minimal impacts on returning to work, may have been over-estimated, particularly if someone recently had the lesion removed. For example, non-melanoma skin cancers are the most common type of cancer in Australia, and in some cases, the cancer is completely removed with a biopsy, or removed by surgical excision [38, 39]. We only included LTHC in our analyses, which was defined in the survey as conditions lasting or expected to last 6-months [20], which should exclude cases in which the only treatment received was complete removal of the lesion.

This paper provides an insight into the economic burden of cancer due to labour force participation, and builds upon the growing body of literature. However, we acknowledge that this is only a small part of the larger burden of cancer, and are currently conducting a larger study on the cost of cancer to the healthcare system and the individual [40]. Future research also should look at the distribution of labour force participation rates across the different types of cancer to ensure equitable access and allocation of resources.

## Conclusion

A large proportion of Australians diagnosed with cancer are of working age, and in general cancer survival rates are improving [3]. Furthermore, in light of the increase of the Australian retirement age, from the current 65 to 67 years by 2023 (increasing by 6 months every 2 years commencing July 2017 [41]), it is reasonable to expect that the cost of lost productivity may increase in the coming years. There is a call for support systems to facilitate return to work for cancer survivors who want to work [42]. For some survivors, returning to work after a cancer diagnosis is an important milestone, both financially and emotionally [33, 43]. Cancer survivors without a tertiary qualification may benefit from additional support. This paper is the first in Australia to estimate the national labour force participation rates of adults of working age with cancer, and the indirect cost of cancer due to lost productivity. This information is valuable when considering how cancer affects patients and society.

## Abbreviations

ABS: Australian Bureau of Statistics
CI: Concentration index
FT: Full-time
GDP: Gross domestic profit
LFP: Labour force participation
LTHC: Long-term health condition
NILF: Not in the labour force
PT: Part-time
SDAC: Survey of Disability, Ageing and Carers

## Footnotes

1 Not in the Labour Force defined as not in the labour force, and have not looked for work in the last 4 weeks, and do not intend on working or looking for work in the future.
